# Zonal-Layered Chondrocyte Sheets for Repairment of Full-Thickness Articular Cartilage Defect: A Mini-Pig Model

**DOI:** 10.3390/biomedicines9121806

**Published:** 2021-11-30

**Authors:** Po-Chih Shen, Cheng-Chang Lu, Shih-Hsiang Chou, Zi-Miao Liu, Shu-Jem Su, Yin-Chun Tien

**Affiliations:** 1 Department of Orthopaedic Surgery, Kaohsiung Medical University Hospital, Kaohsiung 807, Taiwan; shenporch@gmail.com (P.-C.S.); cclu0880330@gmail.com (C.-C.L.); stanelychou@gmail.com (S.-H.C.); 2 Graduate Institute of Medicine, College of Medicine, Kaohsiung Medical University, Kaohsiung 807, Taiwan; 3 Department of Medical Laboratory Science and Biotechnology, School of Medicine and Health Sciences, Fooyin University, Kaohsiung 807, Taiwan; sc096@fy.edu.tw

**Keywords:** cartilage repair, tissue engineering, zonal chondrocyte, cell sheet

## Abstract

The cell sheet technique is a promising approach for tissue engineering, and the present study is aimed to determine a better configuration of cell sheets for cartilage repair. For stratified chondrocyte sheets (S-CS), articular chondrocytes isolated from superficial, middle, and deep zones were stacked accordingly. Heterogeneous chondrocyte sheets (H-CS) were obtained by mixing zonal chondrocytes. The expressions of chondrocytes, cytokine markers, and glycosaminoglycan (GAG) production were assessed in an in vitro assay. The curative effect was investigated in an in vivo porcine osteochondral defect model. The S-CS showed a higher cell viability, proliferation rate, expression of chondrogenic markers, secretion of tissue inhibitor of metalloproteinase, and GAG production level than the H-CS group. The expressions of ECM destruction enzyme and proinflammatory cytokines were lower in the S-CS group. In the mini-pigs articular cartilage defect model, the S-CS group had a higher International Cartilage Repair Society (ICRS) macroscopic score and displayed a zonal structure that more closely resembled the native cartilage than those implanted with the H-CS. Our study demonstrated that the application of the S-CS increased the hyaline cartilage formation and improved the surgical outcome of chondrocyte implication, offering a better tissue engineering strategy for treating articular cartilage defects.

## 1. Introduction

After damage, articular cartilage (AC) undergoes self-repair with great difficulty owing to its avascular nature, aneural structural organization, and low cell-to-matrix ratio [1]. Further, injury to AC often results in the early onset of degenerative joint diseases, and, to date, fully functional cartilage tissue repair remains challenging [2]. At present, despite multiple clinical trials, no drugs have obtained regulatory approval for this indication [3]. By bone marrow stimulating techniques, such as the microfracture, the chondral defect could be fully filled, but compared to natural hyaline cartilage, the regenerated tissue composed of fibrocartilage is deficient in biomechanical and viscoelastic features [4]. Osteochondral autograft/allograft transplantation (OTA) has been demonstrated to be an effective treatment for severe osteochondral defects of the knee. Nonetheless, the limited origin of donors, insufficient mechanical properties, and the morbidity of donor sites are common downsides of the OTA [5]. ACI is an FDA-approved cell-based therapy for the treatment of damaged cartilage and stands as a promising treatment strategy to regenerate the hyaline cartilage [6,7]. In the short-term studies, ACI has shown superior efficiency to the microfracture approach, especially when it is employed to treat larger defects or more severe baseline symptoms [8,9]. Both ACI and OTA are effective to improve the functional outcome in the short-term follow-up, but some series revealed that the patients who received OTA tended to sustain worse conditions than those with ACI [10,11]. Until now, ACI is the still the most widely used cell-based procedure for the regeneration of AC defects, and the evolution of the ACI technique is in progress [12,13,14]. The two-stage operation was considered as the disadvantage of ACI, but it also provides an opportunity to regenerate the zonally relevant AC mimicking the longitudinal histological organization of the native AC. 

The native AC is composed of a matrix that is rich in collagen fibers and proteoglycans (PG), with embedded chondrocytes, including those from the superficial, middle, and deep zones (SZ, MZ, and DZ, respectively), which synthesize the extracellular matrix (ECM) of the cartilage [15,16]. The zonal property of the AC leads to differences within the signaling pathways and provides the tissue with biphasic mechanical properties to counteract compressional loading and shearing force [17,18,19]. Currently, most ACI strategies use mixture populations of chondrocytes that only induce cartilage regeneration with homogenous properties and are not able to effectively regain the zonal organization and functionality of the AC [20,21,22]. Yielding tissue-engineered cartilage that recapitulates the zonal organization of AC is an urgent need and remains a major challenge.

Several approaches attempting to engineer zonally relevant AC have been developed. For example, encapsulating zonal specific chondrocytes in layers of micromass pellets accordingly led to zonal trends in tissue formation. However, the size of the pellets is often too small to revive a large defect, and the zonal variations are spherical rather than depth-dependent. Such features limit a direct application of this approach in clinical treatments. Another approach was a hydrogel-based technique. By encapsulating chondrocytes in a stiffness-gradient hydrogel, with increasing stiffness leading to higher aggrecan expression, it created a zonal-dependent manner, imitating the transition from superficial to deep zone cartilage. However, the hydrogel network often restrains the encapsulated cells, hampers the new matrix deposition, and limits chondrocytes only located in the pericellular regions. This limitation results in the relatively low compressive moduli of resulting cartilage [21,23,24]. A scaffold-based approach may reproduce a perfect layered structure of the native cartilage. Nonetheless, the scaffolds may cause aseptic inflammation and lead to unstable cartilage regeneration, thus seriously limiting the further clinical application [25,26].

We believed that a feasible approach to overcome these setbacks is to use cell sheet technology. The use of cell sheets has been described in regenerative medicine applications, including the regeneration of myocardium [27], cornea [28], and renal tissues [29]. Recently, this technology has also been used to develop chondrocytes sheet for the repair of AC defects. To our knowledge, we are the first study to report the result of using zonal-layer chondrocytes sheet to mimic the depth-dependent organization of cartilage for AC repair. The stratified chondrocytes sheet, which involves a stacking of different zonal chondrocytes sheet derived from AC, is a scaffold-free structure, which mitigates the risk of inflammation and immune rejection. Therefore, the objectives of this study were to: (1) use cell sheet technology to fabricate an engineered cartilage tissue based on either a mixture of chondrocytes or oriented zonal chondrocyte populations, and (2) examine the functional differences between the chondrogenic capabilities and cartilage properties of these two chondrocytes sheet structures.

## 2. Materials and Methods

### 2.1. Cell Preparation and Chondrocytes Sheets Construction

All animal procedures were reviewed and approved by the Institutional Animal Care and Use Committee of Kaohsiung Medical University. Six mini-pigs aged 7–8 months (20–25 kg) were used. The chondral defect, measuring 8 mm in diameter and 5 mm deep, was made in a non-weight-bearing area of the lateral or medial margins of the trochlear groove of the randomly chosen left or right knee (stifle joint) to be used as the source of articular chondrocytes by using a biopsy punch. The chondrocytes from unweighting region were collected to fabricate the cell sheets. Subsequently, the cartilage defects were created in the medial femur condyle where the cell sheets would be implanted. The harvested AC blocks were digested overnight with 0.01% (*w/v*, 0.166 U/mL) collagenase P in medium containing 10% fetal calf serum (FCS) at 37 °C. After then, the freed cells were separated from tissue debris by filtration through a 70-µm nylon cell strainer and collected from the filtrate by centrifugation at 150× *g* for 5 min. The separation of various zonal chondrocytes was performed as previously described with some modifications [30,31]. In brief, chondrocytes were layered on a discontinuous, isotonic Percoll (GE Healthcare) density gradient prepared by weight (densities of 1.015–1.07 g/mL) (Percoll density can be diluted directly to make a final working solution of known density by adding 1.5 M NaCl to 1/10 of the final desired volume and making up to the final volume with distilled water) and centrifuged at 400× *g* for 20 min in a swinging bucket rotor. To fabricate heterogeneous (H-CS) or stratified chondrocyte sheets (S-CS), heterogenous chondrocytes passage 2–3 (P2–P3) or DZ chondrocytes (P2–P3) were harvested according to the methods described above and seeded in a 6-well culture dish at 5 × 10^4^ cells/cm^2^. Cells were cultured until the first layer of chondrocytes reached confluence (about 2–3 days after seeding), and the second layer of chondrocytes (either heterogeneous chondrocytes (P2–P3) or MZ chondrocytes (P2–P3) were seeded on top of the first layer until 100% confluence (about 2–3 days after seeding) was achieved. Then, the third layer of chondrocytes (either heterogeneous chondrocytes (P2–P3) or SZ chondrocytes (P2–P3)) were subsequently seeded on top of the second layer and continuously cultured for an additional 3 weeks. Three weeks later, a thin sheet formed in the cell culture dish. The sheets were collected onto a polyvinylidene difluoride membrane according to the method reported by Yamato et al. [32]. The H-CS and S-CS were harvested and processed for biochemical, histological, and immunofluorescence evaluation. The total cell number for each cell sheet was ranged from 0.89~0.95 × 10^6^ (H-CS) to 1.36–1.46 × 10^6^ (S-CS).

### 2.2. Cell Proliferation and Viability

For detecting cell proliferation rate, the cell numbers of H-CS and S-CS were directly counted. The sheets were digested with TrypLE Express and Collagenase-P, respectively, for 30 min at 37 °C. The dispersed cells were collected and counted using counting chambers. The cell viability was determined by MTT (3-(4,5-Dimethylthiazol-2-yl)-2,5-diphenyltetrazolium bromide) assay.

### 2.3. Gene Expression of Chondrocytes Sheets

Total RNA of chondrocyte sheets was reverse-transcribed by the Thermo Scientific Maxima First Strand cDNA Synthesis Kit (ThermoFisher, Waltham, MA, USA). SYBR qPCR was performed using the SYBR Green qPCR Mix (BIOTOOLS., Co., Ltd. Taipei, Taiwan) kit. Each reaction (20 μL) was run in duplicate and contained 1 μL of cDNA template along with the following primer sequences as listed in Table 1.

Cycling parameters were 95 °C for 15 min to activate DNA polymerase, followed by 40 cycles of 95 °C for 15 s, 60 °C for 20 s, and 72 °C for 30 s. Melting curves were generated at the end of the reaction. Threshold cycles (*C*_t_) for each gene tested were normalized to the housekeeping GAPDH gene value (Δ*C_t_*) and every experimental sample was referred to its control (ΔΔ*C_t_*). Fold change values were expressed as 2^−ΔΔ^*^Ct^*.

### 2.4. Total Glycosaminoglycan (GAG) Quantification and DNA Content Assays

Total GAG content of chondrocyte sheets was determined using DMMB (Polysciences, Warrington, PA, USA) as previous studies described [33]. DNA content of chondrocyte sheets was measured using Hoechst 33258 dye (ThermoFisher, Waltha, MA, USA). Briefly, 10 μL of the digested chondrocytes sheets was mixed with 200 μL of Hoechst dye solution (0.7 μg/mL). Fluorescence measurements were performed with an excitation wavelength of 340 nm and an emission wavelength of 465 nm. The calf thymus DNA was used as the standard curve. The GAG content was normalized to the amount of DNA measured per sample.

### 2.5. Measurement of Humoral Factors

Condition media from H-CS and S-CS were collected and centrifuged at 12,000× *g* for 10 min to remove cell debris. The concentrations of transforming growth factor beta-1 (TGF-β1), tissue inhibitor of metalloproteinases-3 (TIMP-3), tissue inhibitor of metalloproteinases-1 (TIMP-1), matrix metalloproteinase-3 (MMP3), and matrix metalloproteinase-13 (MMP13) were measured using enzyme-linked immunosorbent assay (ELISA) kits. Measurements were repeated at least 3 times for each donor, and averages were used.

### 2.6. Immunofluorescence Assay

Frozen sections of triple-layered cell sheets were fixed and frozen by using OCT compound. The sections were incubated with Col2a1 primary antibodies (Proteintech,15943-1-AP, 1:100 dilution), Acan (Proteitech, 13880-1-AP), MMP-3 (Proteintech 66338-1-Ig), MMP-13 (Proteintech, 18165-1-AP), ADAMTS-4 (ABclonal, A2525) ADAMTS-5 (Abclonal, A2836), and a secondary antibody (anti-Rabbit IgG (H+L)-TAMRA and anti-Mouse IgG (H+L)-FAM). The cell nuclei were stained with 4′-6-diamidino-2-phenylindole (DAPI). The samples were then observed and photographed observed under a high-quality fluorescence microscope.

### 2.7. In Vivo Implantation of Chondrocyte Sheets

After the H-CS and S-CS were prepared, the sheets were implanted into the same animal from which the cells were derived. Three groups, each containing 6 mini-pigs (aged 7–8 months; sex mature age: 3–5 months), were preconditioned by a biopsy punch to introduce two chondral defects, measuring 8 mm in diameter and 5 mm deep, in the animals’ medial femoral condyle. The three groups were then treated as follows: the control group (n = 6), without any filler cells; the H-CS group (n = 6), which was filled with the H-CS (constructed by mixing zonal chondrocytes); and the S-CS group (n = 6), filled with the S-CS (constructed by zonal chondrocytes in histological organization).

### 2.8. Macroscopic Evaluation by ICRS Scoring System

The mini-pigs in each group were sacrificed at 12 weeks after the implantation and the cartilage was harvested. The defect sites were photographed and scored by using the International Cartilage Repair Society (ICRS) scoring system. The total score ranges from 0 to 12 and includes scores from three categories: degree of defect repair, integration to the border zone, and macroscopic appearance [34].

### 2.9. Histology and Immunohistochemical Examination 

The harvest cartilage pieces were fixed in 4% paraformaldehyde, dehydrated in a graded ethanol, and then embedded in paraffin. Specimens were stained with hematoxylin–eosin (H&E) stain, Safranin-O stain, and Alcian blue stain. Immunohistological analysis was also performed. Col2a1 primary antibodies, ACAN and Col10a1, and the secondary antibody (DAKO) were successively subjected to immunohistological assay. The samples were then observed and photographed under a high-quality microscope.

### 2.10. Histological Grading Score for the Assessment of Cartilage Repair

Tissue sections were evaluated using the histological grading score described by Mankin [35], which was modified as described previously [28]. The total score ranges from 0 to 14 and includes scores from four categories: cartilage structure, cellular abnormality, matrix staining, and tidemark integrity.

### 2.11. Statistics

All analyses were performed using the Statistical Package for Social Sciences (Version 19.0, SPSS Inc., Chicago, IL, USA). Each sample was independently tested at least three times and the values were presented as the mean ± standard deviation (SD). The statistically significant difference between the groups was analyzed using *t-test* or one-way ANOVA. Statistical significance was indicated at *p* < 0.05.

## 3. Results

### 3.1. Functional Properties of SZ, MZ, and DZ Chondrocytes

**Figure 1:**
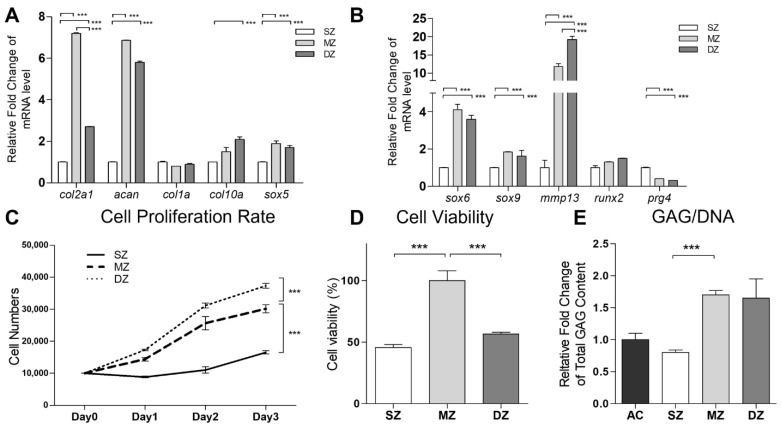
**Functional properties of separation of superficial zone (SZ), middle zone (MZ), and deep zone (DZ) of chondrocytes.** The porcine articular chondrocytes by discontinuous Percoll gradient. (**A**,**B**) The mRNAs of *col2a1*, *Acan*, *col1a1*, *col10a1*, *sox5*, *sox6*, *sox9*, *mmp13*, *runx2*, *prg4*, and *gapdh* among SZ, MZ, and DZ of chondrocytes were analyzed by real time PCR. The modulation of mRNA expression was expressed relative to the SZ after normalization to the *gapdh* signal. (**C**) Cell proliferation rates of SZ, MZ, and DZ of chondrocytes were determined by counting the cell numbers for 3 days. (**D**) The SZ, MZ, and DZ of chondrocytes were cultured in the monolayer for 72 h. The cell viability was then detected using an MTT assay. (**E**) Total glycosaminoglycan (GAG) content was measured in monolayer culture. (***, *p* < 0.001).

The chondrocytes in each zone (SZ, MZ, and DZ chondrocytes) of the AC, with densities varying in the range 1.015–1.070 g/mL, showed differences in size. This made their fractionation using a discontinuous Percoll gradient possible. Specifically, the largest cells with the lowest cell density in the uppermost fraction were considered as DZ chondrocytes, the smallest ones in the lowest layer as SZ chondrocytes, and the rest in the middle as MZ chondrocytes (Supplementary Figure S1A). To verify whether the density gradient strategy separated the chondrocytes efficiently, we analyzed the mRNA expression of *col2a1*, *acan*, *col2a1*, *col10a1*, *sox5*, *sox6*, *sox9*, *mmp13*, *runx2*, and *prg4* (Figure 1A,B). It was observed that the genes involved in chondrocyte differentiation, including *col2a1*, *acan*, *sox5*, *sox6*, and *sox9*, were more upregulated in the MZ chondrocytes than in the DZ and SZ chondrocytes. The expression levels of *col10a1*, *mmp13*, and *runx2* were higher in the DZ chondrocytes. The SZ chondrocytes showed the highest level of *prg4* expression and were characterized by a decrease in the cell proliferation rate (Figure 1C) and cell viability (Figure 1D), with the synthesized cartilage matrix containing lower levels of PGs (Supplementary Figure S1B) and GAG (Figure 1E) compared with those corresponding to MZ and DZ chondrocytes.

### 3.2. Cell Viability, Cell Proliferation, and Expression of Chondrogenic Markers in H-CS and S-CS

**Figure 2:**
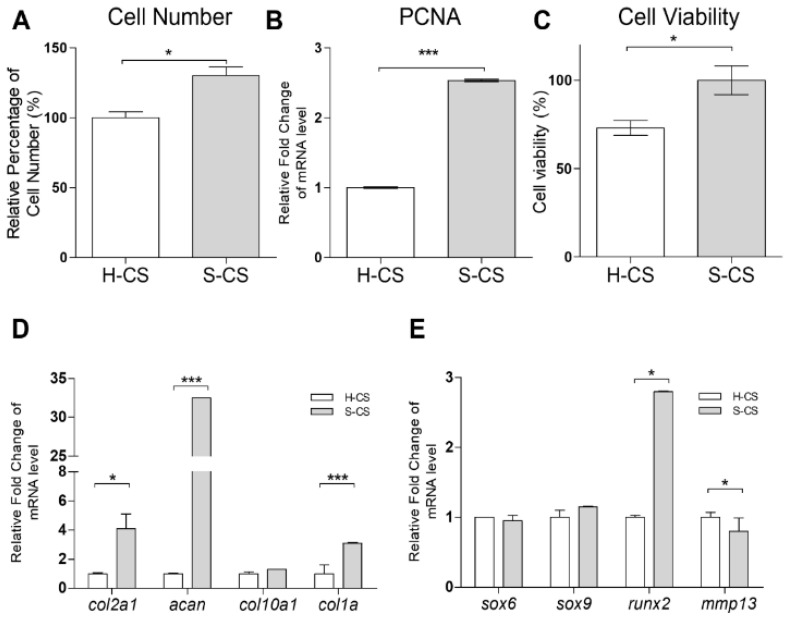
**Comparison the cell proliferation, viability, and chondrogenic-related gene expression of heterogeneous chondrocyte sheets (H-CS) and stratified chondrocyte sheets (S-CS).** (**A**) Cell proliferation rates of different sheets were determined by counting the cell numbers using a hemacytometer. (**B**) The mRNA expressions of *pcna* in the H-CS and S-CS were detected by qRT-PCR. (**C**) The cell viability of was detected using an MTT assay * compared with the H-CS group. (**D**,**E**) The mRNAs of *col2a1*, *acan*, *col10a1*, *col1a*, *sox5*, *sox6*, *sox9*, *runx2*, *mmp13*, and *gapdh* of H-CS and S-CS were analyzed by qRT-PCR. The modulation of mRNA expression was expressed relative to the H-CS after normalization to the *gapdh* signal. (*, *p* < 0.05, ***, *p* < 0.001).

After 3 weeks of culturing in vitro, the cells were harvested and counted. The mean number of cells in the S-CS (range, 1.36–1.46 × 10^6^) was significantly higher than that in the H-CS (range, 0.89–0.95 × 10^6^) (Figure 2A). Additionally, the transcriptional analysis of proliferating cell nuclear antigen (*pcna*) showed a 2.5-fold increase in the expression of the antigen in the S-CS compared with that in the H-CS (Figure 2B). An MTT assay also showed a slight increase in the average live cell percentage in the S-CS (Figure 2C). Comparing the cartilage-formation capacities, the mRNA expression of *col2a1* and *acan* in the S-CS (S-CS vs. H-CS, *col2a1*, 4.8-fold; *acan*, 30-fold) increased significantly. Conversely, the mRNA expression of *mmp13* showed a more significant decrease in the S-CS than in the H-CS (S-CS vs. H-CS, *mmp13*, 0.8-fold) (Figure 2D,E).

### 3.3. Secretion of ECM Destructive Enzymes in H-CS and S-CS

**Figure 3:**
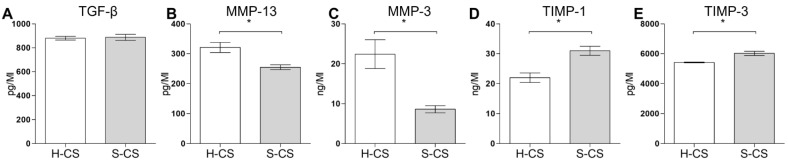
**Comparison of the concentrations of humoral factors secreted by heterogeneous chondrocyte sheets (H-CS) and stratified chondrocyte sheets (S-CS).** To determine the humoral factors’ expression levels, the cell culture medium was collected at 72 h after triple layer sheets were fabricated. The protein levels of (**A**) TGF-β1, (**B**) MMP13, (**C**) MMP3, (**D**) TIMP-1, and (**E**) TIMP-3 were measured as Material and Methods described. Data are presented as bar with median concentration. (*, *p* < 0.05).

To investigate the protein levels of TGF-β, MMP-3, MMP-13, TIMP-1, and TIMP-3 in the H-CS and S-CS, supernatants were collected from the cell sheet cultures and subjected to ELISA. The concentrations of the humoral cytokines secreted by these two sheets are summarized in Figure 3, from which it is evident that the S-CS produced higher concentrations of TIMP-3 (6100–6200 pg/mL) and TIMP-1 (31–33 ng/mL) than the H-CS (TIMP-3, 5320–5470 pg/mL; TIMP-1, 22–23 ng/mL; Figure 3D,E). By contrast, the H-CS produced higher concentrations of MMP-13 (320–340 ng/mL) and MMP3 (22–26 ng/mL) than the S-CS (MMP-13, 260–275 ng/mL; MMP3, 7–8 ng/mL; Figure 3B,C). However, with respect to TGF-β1 concentrations, the H-CS and S-CS showed no significant difference (Figure 3A).

### 3.4. Pro-Inflammatory Cytokine Gene Expression in H-CS and S-CS 

**Figure 4:**
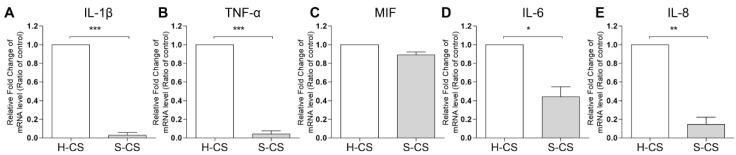
**Comparison of the pro-inflammatory cytokine expression in heterogeneous chondrocyte sheets (H-CS) and stratified chondrocyte sheets (S-CS).** To determine the proinflammatory gene expression levels, the total RNA was extracted at 3 weeks after triple layer sheets were fabricated. The mRNA levels of (**A**) IL1-β, (**B**) TNF-α, (**C**) MIF, (**D**) IL-6, and (**E**) IL-8 were measured by qRT-PCR. (*, *p* < 0.05, **, *p* < 0.01, ***, *p* < 0.001).

Reportedly, the expression of pro-inflammatory cytokines, such as IL-1β and TNF-α, in transplanted tissues can have negative effects on the clinical outcomes of ACI treatment [17,36]. In this study, the mRNA expression of *IL-1β*, *TNF-α*, *IL-6*, *IL-8*, and *MIF* in the H-CS and S-CS was determined via qRT-PCR. As shown in Figure 4, the transcript-level expression of *IL-1β*, *TNF-α*, *IL-6*, and *IL-8* in the S-CS was lower than that in H-CS (S-CS vs. H-CS, *IL-1β*, 0.03-fold; *TNF-α*, 0.01-fold; *IL-6*, 0.4-fold; and *IL-8*, 0.2-fold).

### 3.5. Comparison of Matrix Production Abilities and Immunohistochemical Analyses of H-CS and S-CS

**Figure 5:**
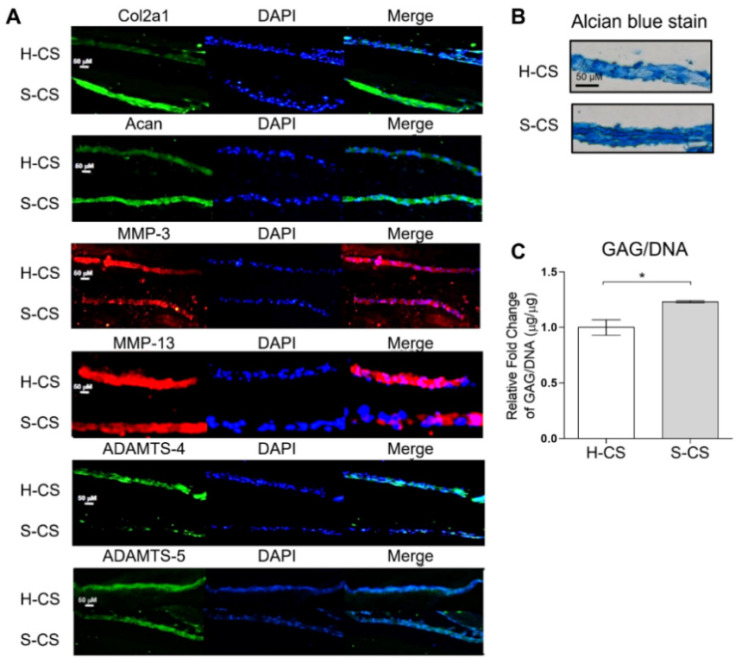
**Microscopic images of histological section.** Image of heterogeneous chondrocyte sheets (H-CS) and stratified chondrocyte sheets (S-CS). (**A**) Immunofluorescence staining for Col2a1, Acan, MMP-3, MMP-13, ADAMTS-4, and ADAMTS-5. (Scale bar = 50 μm) (**B**) Alcian blue stain of H-CS and S-CS. (**C**) Total glycosaminoglycan (GAG) content of H-CS and S-CS. (*, *p* < 0.05).

Western blotting, Alcian blue staining, and immunofluorescence were performed to investigate the chondrogenic properties of the H-CS and S-CS. Immunofluorescence revealed increased Col2a1 levels as well as stronger Acan staining in defects implanted with the S-CS than in those implanted with the H-CS. Conversely, defects implanted with the S-CS exhibited reduced expression of MMP-3, MMP-13, ADAMTS-4, and ADAMTS-5 compared to those implanted with the H-CS (Figure 5A). As shown in Figure 5B, Alcian blue staining revealed a deeper blue color in defects implanted with the S-CS than in those implanted with the H-CS, indicating that the S-CS had better ECM production capacity (Figure 5C).

### 3.6. In Vivo Repair Evaluation Based on Gross Appearance and Histology

**Figure 6:**
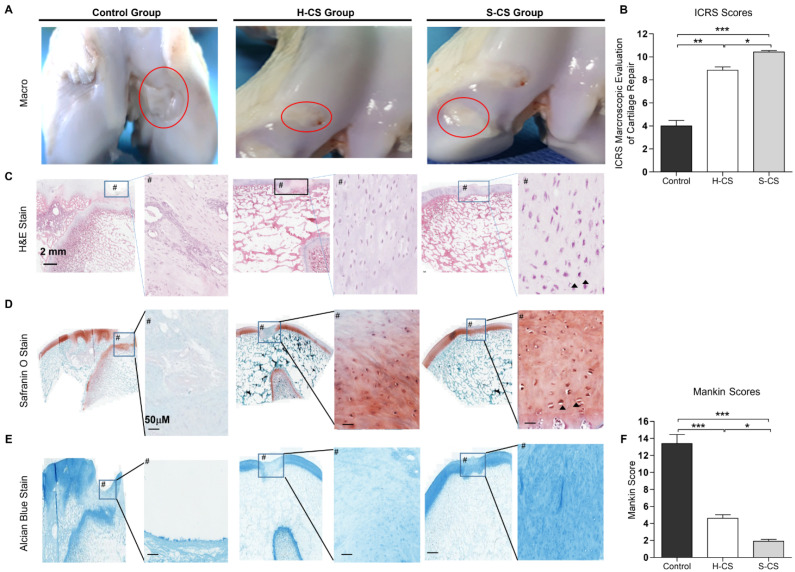
**Macroscopic and histological observation of regenerated cartilage.** (**A**) Photographs represent porcine knee articular defect healing in the control, heterogeneous chondrocyte sheets (H-CS), and stratified chondrocyte sheets (S-CS) groups at 12 weeks after surgery. The red circles indicate the original defect margin. (**B**) International Cartilage Repair Society (ICRS) macroscopic assessment scores of repaired cartilages at 12 weeks. Data are presented as mean ± SD. (**C**) H&E staining. Boxes in left panels represent magnified area shown in right panels. (**D**) Staining results of Safranin O/Fast green and (**E**) Alcian blue in low-powered (left) and high-powered view (scale = 100 μM) (right image, #). Arrowheads indicate the lacunae. (**F**) Sections were scored with Mankin score. (*, *p* < 0.05, **, *p* < 0.01, ***, *p* < 0.001).

At twelve weeks after the implantation surgery, articular joint samples were harvested for gross and histological evaluation, which were based on the mean gross grading with respect to the defect coverage degree, neocartilage color, the integration of the border zone, and surface smoothness. The articular joint samples resulting from both the H-CS and S-CS showed improved regeneration of the osteochondral defects compared with the control group (Figure 6A). Overall, in the animals that were implanted with the S-CS, the defects were completely covered with the reparative tissue, whereas the osteochondral defects in the other two groups were only partially covered (Figure 6A). Additionally, in the group implanted with the S-CS, the boundaries between the implanted tissue and the native tissue were unclear and the newly formed tissue integrated almost completely with the adjacent normal tissues (Figure 6A). Further, the articular surfaces in the S-CS group appeared more intact and smoother and resembled normal articular tissue than those in the H-CS group (Figure 6A). Quantitatively, the ICRS macroscopic scores corresponding to the H-CS (9 ± 0.3) and the S-CS (10.5 ± 0.1) were markedly higher than those corresponding to the control (4 ± 0.5) (Figure 6B). To observe the differences between the cellular structures and matrix compositions resulting from the two implantation groups, microscopic histological examinations were performed using HE, Safranin-O, and Alcian blue staining (Figure 6C–E). The defects in the control group were characterized by reduced cellular distribution, surrounded by loose connective tissue resembling fibrous tissue, and showed very weak staining for Alcian blue and Safranin-O. Compared with the control groups, the defects implanted with H-CS showed a more even distribution of the chondrocytes as well as increased Alcian blue and Safranin-O staining intensity. Meanwhile, in the S-CS groups, the neocartilage displayed a zonal structure that closely resembled that of the native cartilage; the cells were densely distributed in the SZ and their PG content (stained with Safranin-O) was lower. In the MZ of this stratified sheet, an increase in the PG content with depth was observed, and the chondrocytes appeared rounded and more sparsely populated than those in the SZ. Moreover, lacunae were clear (Figure 6D and Supplementary Figure S2). Further, in the DZ, the chondrocytes were arranged in columns and exhibited cartilage-specific lacunae (Supplementary Figure S2). In sum, the S-CS group demonstrated the strongest Alcian blue and Safranin-O staining intensities. Finally, using the Mankin histological scoring system [35], a relative-quantitative evaluation was performed to determine the quality of the regenerated cartilage tissues. The scores obtained for the control, H-CS, and S-CS groups were 13.2, 4.3, and 1.8, respectively. This evaluation revealed a significantly improved histological score for the S-CS group compared with those in the other two groups (Figure 6F).

**Figure 7:**
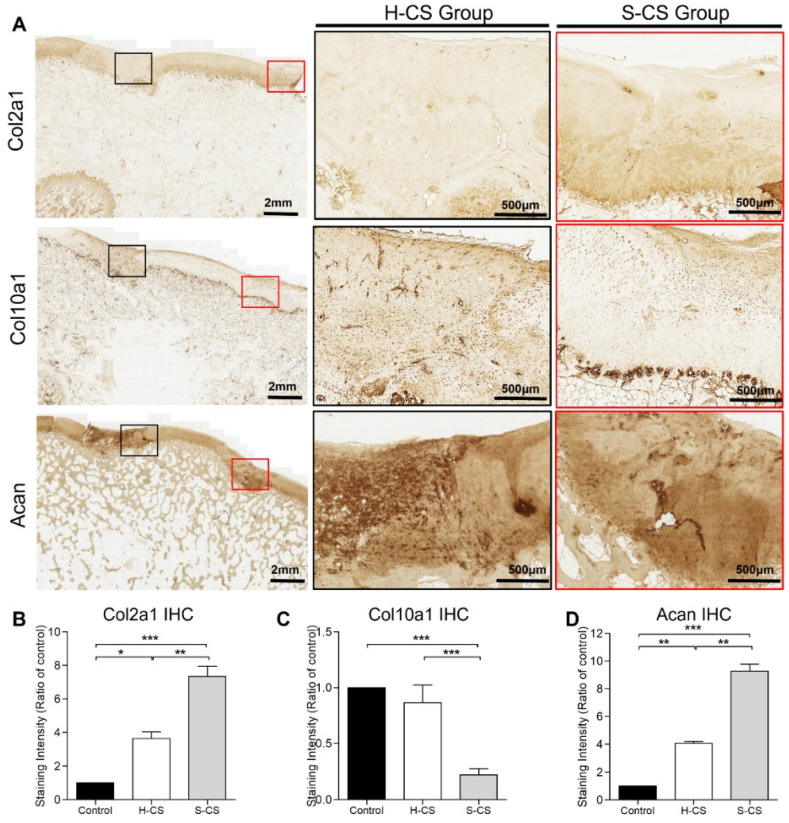
**Immunohistochemical staining of regenerated cartilages.** (**A**) The representative images of immunohistochemical staining for Col2a1, Col10a1, and Acan. The black and red boxes indicate the defect implanted with the heterogeneous chondrocyte sheets (H-CS) and the stratified chondrocyte sheets (S-CS), respectively. The low-powered view is as indicated in left panel (2 mm), and the high-powered views inside each box are present at right side (500 μm). The quantitative analysis of the integrated optical density (IOD) for (**B**) Col2a1 staining, (**C**) Col10a1 staining, and (**D**) Acan staining of the three groups at 12 weeks after surgery. (*, *p* < 0.05, **, *p* < 0.01, ***, *p* < 0.001).

To further characterize the composition of the neocartilage, it was necessary to distinguish between the mature cartilage and hypertrophic cartilage. Thus, we performed immunohistochemistry for Col2a1, Acan, and Col10a1 proteins. As shown in Figure 7, neocartilage corresponding to the H-CS and S-CS groups showed positive results for Col2a1 and Acan staining, while the control group showed no staining. Additionally, Col2a1 was expressed in the ECM of the regenerated tissues within the defect, whereas Acan was localized both intracellularly and within the ECM. Like the integrated optical density (IOD) measurement results, the S-CS group presented stronger Col2a1 and Acan staining than the H-CS group (Figure 7A,B,D). Conversely, the neocartilage resulting from the H-CS showed stronger staining for Col10a1 (Figure 7A,C) than that resulting from the S-CS, indicating that the neocartilage of the S-CS group resembled the hyaline cartilage, as evidenced by the abundant deposition of PG and Col2a1 and the scarcity of Col10a1.

## 4. Discussion

In this study, the discontinuous Percoll gradient centrifugation method was used to separate the zonal chondrocytes accordingly. The sizes of the cells in each zone were also confirmed before the in vitro expansion (P0). As shown in Figure 1A, the uppermost fraction—which consisted of larger cells and higher PG concentrations (detected within the P1–P2)—was primarily derived from the DZ of the AC as previously reported [37,38]. Conversely, the cells from the lowest fraction, which were the smallest, had low PG concentrations, and proteoglycan 4 (PRG4) showed higher expression, were derived from the SZ. These cells also showed slower division than those derived from the MZ and DZ [39]. Further, ECM marker analysis (within the P1–P2) showed that significantly higher *acan* and *col2a1* expression levels were associated with the middle and uppermost fractions (MZ and DZ fractions, respectively) (Figure 1E). These results are consistent with those reported in previous studies, in which dissection methods as well as the cell size-based inertial spiral microchannel technique were used to separate the SZ, MZ, and DZ chondrocytes from full-thickness cartilage blocks [40,41]. However, the purity still needed to improve in the zonal chondrocytes. Recently, strategies that involve the use of microarray datasets to analyze the transcriptome profile of the laser capture microdissection (LCM) zones of mouse AC have been developed to identify the molecular markers in each zone [42]. Nevertheless, the suitable cell surface marker identified in small animals might not apply in different species. Additionally, many established AC markers, such as Cilp, Tnn, Cxcl14, and Pcp4, were also not appropriate for cell sorting in large animals. Hence, for isolating the different layer chondrocytes of the AC in large animals more precisely, the appropriate cell surface markers need to be further clarified. Furthermore, in the present study, we have not yet conducted experiments using chondrocyte subpopulations derived from sheet tissue sections to analyze the ability of zonal chondrocytes to maintain their different biosynthetic activities following weeks of culturing. However, another study indicated that, after several weeks of culturing, encapsulating individual layers of chondrocytes by hydrogel showed histologic features that were similar to those of the native AC [40]. Further, it also demonstrated that high-density chondrocyte cultures (as in this study) showed superior capacities to maintain the phenotypic stability of chondrocytes compared to low-density chondrocyte cultures [43,44], and the chondrocyte hypertrophy and dedifferentiation did not occur.

The in vitro experiments conducted in the present study also revealed that the S-CS may be more advantageous than the H-CS with respect to tissue engineering applications. This conclusion is due to the fact that, when compared with the H-CS, the S-CS demonstrated increased cell viability, proliferation rates, cartilage-specific marker mRNA and protein levels, and GAG contents, as well as decreased ECM-destructive proteases. In addition, the S-CS exhibited reduced levels of pro-inflammatory cytokines, including IL-6, IL-8, and TNF-α, compared to the H-CS (Figure 4). Reportedly, the expression of pro-inflammatory cytokines, including IL-1β, IL-6, IL-8, and TNF-α, in implanted chondrocytes significantly influences the clinical outcomes of ACI [36,45,46] Therefore, the ability to regulate the expression of principal pro-inflammatory cytokines, such as IL-1β and TNF-α, observed in S-CS possibly resulted in an improved ACI performance. However, the molecular mechanism by which these S-CS slices brought about relatively lower expression of pro-inflammatory cytokines than the H-CS slices is still unclear. Based on our results, we reasoned that the S-CS that mimicked the zonal organization of AC, and such multiple layers of intracellular crosstalk between the different chondrocytes, benefited the signal transmission. For example, the recovery of the SZ was required to establish proper signaling to the underlying tissue and to ensure the modulation of the proliferative rate as well as the biosynthetic activities of the DZ chondrocytes. Furthermore, MZ chondrocytes enhanced the expression amount of the PRG-4 secreted by the SZ chondrocytes [21,47]. In addition, in damaged cartilage, the elevated levels of cytokines, including ILs and TNFs, which have been shown to enhance MMPs levels, cell death, and NO production, subsequently inhibited the PG synthesis and contributed to the diseased state of the tissue. In this study, we observed that the S-CS expressed relatively lower levels of pro-inflammatory cytokines, including IL-1β and TNF-α, which might explain the observed increase in cell viability and PG expression, as well as the lower MMPs in the S-CS. Further, a recent study has found that the cell sheet technique (using mixtures of different zones of chondrocytes) exhibited a relatively lesser inflammatory response than the chondrocyte/scaffold in an in vivo porcine model [48]. In the current study, we demonstrated that S-CS has a superior anti-inflammatory response to H-CS.

Related studies have shown that using zonal chondrocyte subpopulation to fabricate the mimetic native organization of AC might improve the functionality of the resulting graft [20,21,42,49]. However, using zonal chondrocytes for the reconstruction of the functional tissue has not yet been achieved, and no long-term in vivo studies have been reported. For example, seeding chondrocyte subpopulations sequentially with SZ and MZ into alginate to fabricate stratified implants in mini-pig models showed features of early repair response after one week of implantation. However, the implanted construct did not maintain a stratified organization within the defect after one week in vivo [50]. Additionally, in a recent study, a bi-layered fibrin gel construct consisting of MZ/DZ chondrocytes overlaid with SZ chondrocytes was applied in a small rat osteochondral defect animal model, and it was observed that the two distinct layers were preserved at the cartilage defect site for at least two weeks post-implantation [49]. In the current study, using a porcine osteochondral defect model, we delivered the S-CS to bring about the repair of damaged cartilage and compared the outcomes with those corresponding to the delivery of the H-CS. The results of the histological scoring showed that the implantation of separated zonal chondrocytes as tri-layered sheets resulted in the formation of neocartilage with a hyaline-like and native cartilage-characteristic zonal structure after twelve weeks of implantation (Figure 6 and Figure 7). Additionally, this approach yielded cartilage with better quality; hence, it represents a significant advancement compared with the implantation of un-layered chondrocytes or no implantation. Particularly, when the H-CS was used, the resulting neocartilage did not mimic the native zonal architecture after twelve weeks of implantation.

The present study had several limitations. First, the exact mechanisms by which the layered sheets retain chondrogenic properties were not clarified. Therefore, in the future, it would be necessary to examine the activation of the intracellular signaling pathways and evaluate the key extracellular bioactive factors in the layered sheets. Second, the mechanical compressive modulus of the tri-layered implantation in our study was not examined; thus, further studies are needed in this regard. Third, the cell sheets were fabricated from pigs with ages in the range 7–8 months, which relatively corresponds to young human adults, and the cartilage tissue was collected in bulk from areas that appeared normal. Conversely, patients who undergo total knee arthroplasty (TKA) are generally aged between 50 and 80 years, and, during such treatment interventions, the cartilage tissues are collected from the non-loading regions of knee cartilage. Hence, comparisons must be made carefully given that the quality of the TKA sheets may be inferior to that of the sheets used in this experimental study. Additionally, we had to utilize a two-stage surgical approach, first to collect the healthy cartilage and second to transplant the chondrocyte sheets. The harvesting of autologous cells requires the sacrifice of healthy cartilage at a non-loading site of the knee, which comprises a limited area. Therefore, the application of this strategy is limited. Further, we observed the prospective outcomes only for a period of twelve weeks. This implies that longer-term observations are necessary to fully evaluate the outcomes of this therapy. Finally, we did not evaluate the remodeling of the subchondral bone of each pig after implantation with or without the cell sheets. It has been suggested that the change in subchondral bone stiffness may affect the AC regeneration [51]. The connection between chondrocytes sheet implantation and subchondral bone remodeling requires further investigation.

In conclusion, this is the first study that demonstrated that the zonal subpopulation chondrocytes that generated S-CS had better cell proliferation ability, cartilage-specific marker expression, and ECM production. The S-CS also had less expression of the pro-inflammatory cytokine and ECM destruction enzyme. The in vivo investigation using the mini-pig model also revealed that the S-CS had promotion in the ICRS macroscopic scores and histological findings. Our approach provides a relatively straightforward method to recapitulate the zonal structure of AC and represents a significant advancement in the current chondrocyte-based AC regeneration.

## Figures and Tables

**Table 1:** Primer used in the present study.

Gene	Forward Primer 5′-3′	Reverse Primer 5′-3′
*col2a1*	ACTCCTGGCACGGATGGTC	CTTTCTCACCAACATCGCCC
*aggrecan*	CCCAACCAGCCTGACAACTT	CCTTCTCGTGCCAGATCATCA
*col10a1*	TGAACTTGGTTCATGGAGTGTTTTA	TGCCTTGGTGTTGGATGGT
*sox5*	GGCCAAGCAGCAGCAAGAACAG	AGCTGAAGCCTGGAGGAAGGAG
*sox6*	CAGCCCTGTCAGTCTGCCTAACA	GCATCTTCCGAGCCTCCTGAATAGC
*sox9*	GGCAATCCCAGGGTCCACCAAC	TGGTCGAACTCGTTGACGTCGAAG
*mmp13*	ACCCAGGAGCCCTCATGTTTCC	CAGGGTTTCTCCTCGGAGACTG
*runx2*	CCAGACCAGCAGCACTCCATAC	GGGAACTGCTGTGGCTTCCATC
*prg4*	CTCCCAAGGAGCAGCTTCTAC	GGTGGTGGGAGCTGGTTCCTTG
*pcna*	GCGCCTGGTCCAGGGC	TCACGCCCATGGCCAAATTGC
*IL-1*	GTACATGGTTGCTGCCTGAA	CTAGTGTGCCATGGTTTCCA
*IL-6*	GGCAGAAAACAACCTGAACC	GTGGTGGCTTTGTCTGGATT
*IL-8*	TAGGACCAGAGCCAGGAAGA	CAGTGGGGTCCACTCTCAAT
*TNF*	ACTGCACTTCGAGGTTATCG	GCTGGTTGTCTTTCAGCTTC
*MIF*	CGTGCGCCCTTTGCAGTCTG	TGGCCGCGTTCATGTCGTAG

## Data Availability

All data presented in this study are available upon reasonable request from the corresponding author.

## Supplementary Materials

The following are available online at https://www.mdpi.com/article/10.3390/biomedicines9121806/s1. Figure S1: Separation of porcine articular chondrocytes by discontinuous Percoll gradient. (A) Photomicrographs showing the different sizes of the chondrocyte populations after Percoll gradient separation. (B) The superficial zone (SZ), middle zone (MZ), and deep zone (DZ) of chondrocytes were stained with Alcian blue (×100 magnification). Figure S2: Histological observation of regenerated cartilage. Images of safranin O staining of the cartilage defect implanted with stratified chondrocyte sheets (S-CS). The neocartilage displayed more closely resembled the native cartilage. In the superficial zone, cells were densely distributed, and proteoglycan content (stained with Safranin O) was lower. In the middle zone, the proteoglycan content was increased, and lacunae were clearly observed (solid line box). In the deep zone, chondrocytes were arranged in columns (dash line box).
