# Supplementary material for: Zonal-Layered Chondrocyte Sheets for Repairment of Full-Thickness Articular Cartilage Defect: A Mini-Pig Model
Source: Biomedicines. 2021 Nov 30;9(12):1806. doi: 10.3390/biomedicines9121806 (PMC8698967; doi:10.3390/biomedicines9121806)
Supplement: Supplementary file 1 [file biomedicines-09-01806-s001.zip › biomedicines-1468721-supplementary.pdf]

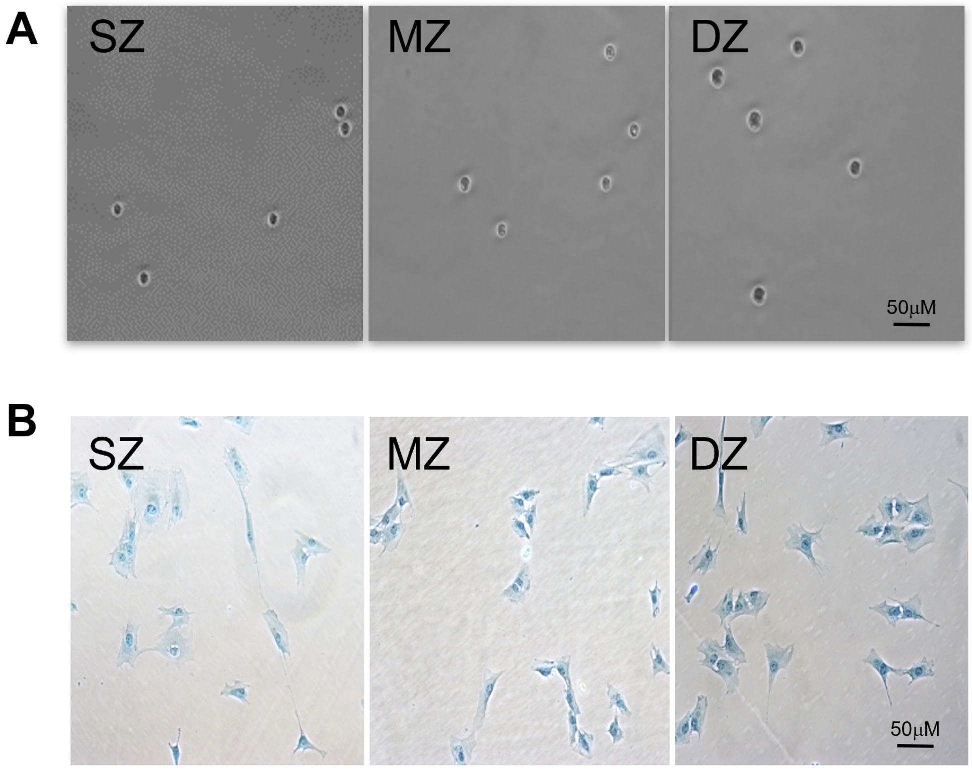

Figure S1. Separation of porcine articular chondrocytes by discontinuous Percoll gradient. (**A**) Photomicrographs showing the different sizes of the chondrocyte populations after Percoll gradient separation. (**B**) The superficial zone (SZ), middle zone (MZ), and deep zone (DZ) of chondrocytes were stained with Alcian blue (×100 magnification).

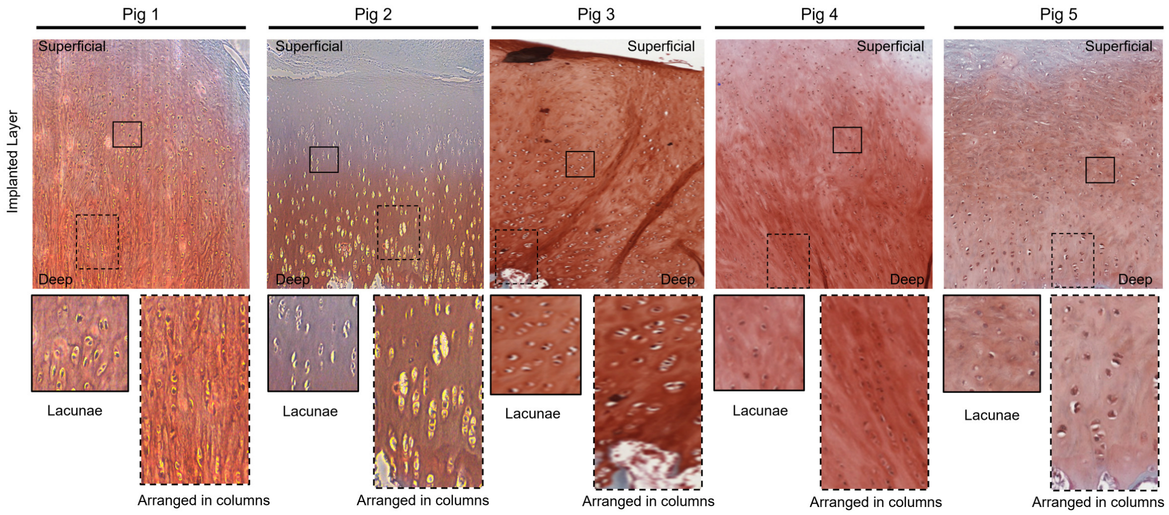

Figure S2. Histological observation of regenerated cartilage. Images of safranin O staining of the cartilage defect implanted with stratified chondrocyte sheets (S-CS). The neocartilage displayed more closely resembled the native cartilage. In the superficial zone, cells were densely distributed, and proteoglycan content (stained with Safranin O) was lower. In the middle zone, the proteoglycan content was increased, and lacunae were clearly observed (solid line box). In the deep zone, chondrocytes were arranged in columns (dash line box).
